# NK Cell Patterns in Idiopathic Inflammatory Myopathies with Pulmonary Affection

**DOI:** 10.3390/cells10102551

**Published:** 2021-09-27

**Authors:** Marc Pawlitzki, Christopher Nelke, Leoni Rolfes, Rebecca Hasseli, Stylianos Tomaras, Eugen Feist, Anne Schänzer, Saskia Räuber, Liesa Regner, Corinna Preuße, Yves Allenbach, Olivier Benveniste, Heinz Wiendl, Werner Stenzel, Sven G. Meuth, Tobias Ruck

**Affiliations:** 1 Department of Neurology with Institute of Translational Neurology, University Hospital Münster, 48149 Münster, Germany; ChristopherJannik.Nelke@med.uni-duesseldorf.de (C.N.); liesa.regner@med.uni-duesseldorf.de (L.R.); heinz.wiendl@ukmuenster.de (H.W.); 2 Department of Neurology, University Hospital Düsseldorf, 40225 Düsseldorf, Germany; Leoni.Rolfes@med.uni-duesseldorf.de (L.R.); saskia.raueber@med.uni-duesseldorf.de (S.R.); SvenGuenther.Meuth@med.uni-duesseldorf.de (S.G.M.); 3 Department of Rheumatology and Clinical Immunology, Kerckhoff-Klinik GmbH, 61231 Bad Nauheim, Germany; R.hasseli@kerckhoff-Klinik.de; 4 Department of Rheumatology, Helios Fachklinik Vogelsang-Gommern, 39245 Gommern, Germany; stylianos.tomaras@helios-gesundheit.de (S.T.); eugen.feist@helios-gesundheit.de (E.F.); 5 Institute of Neuropathology, Justus Liebig University Giessen, 35392 Giessen, Germany; Anne.Schaenzer@patho.med.uni-giessen.de; 6 Department of Neuropathology, Charite Medical Faculty Berlin, 13353 Berlin, Germany; corinna.preusse@charite.de (C.P.); werner.stenzel@charite.de (W.S.); 7 Department of Clinical Immunology and Internal Medicine, Sorbonne University, APHP, Pitié Salpêtrière Hospital, 75013 Paris, France; yves.allenbach@ghps.fr (Y.A.); olivier.benveniste@aphp.fr (O.B.); 8 INSERM, Myology Research Centre, 75013 Paris, France

**Keywords:** inflammatory myopathy, myositis, interstitial lung disease, antibodies, natural killer cells

## Abstract

Background: Pulmonary affection (PA) is associated with a substantial increase in morbidity and mortality in patients with idiopathic inflammatory myopathies (IIM). However, the underlying immune mechanisms of PA remain enigmatic and prompt deeper immunological analyses. Importantly, the Janus-faced role of natural killer (NK) cells, capable of pro-inflammatory as well as regulatory effects, might be of interest for the pathophysiologic understanding of PA in IIM. Methods: To extend our understanding of immunological alterations in IIM patients with PA, we compared the signatures of NK cells in peripheral blood using multi-color flow cytometry in IIM patients with (*n* = 12, of which anti-synthetase syndrome = 8 and dermatomyositis = 4) or without PA (*n* = 12). Results: We did not observe any significant differences for B cells, CD4, and CD8 T cells, while total NK cell numbers in IIM patients with PA were reduced compared to non-PA patients. NK cell alterations were driven by a particular decrease of CD56^dim^ NK cells, while CD56^bright^ NK cells remained unchanged. Comparisons of the cell surface expression of a large panel of NK receptors revealed an increased mean fluorescence intensity of NKG2D^+^ on NK cells from patients with PA compared with non-PA patients, especially on the CD56^dim^ subset. NKG2D^+^ and NKp46^+^ cell surface levels were associated with reduced vital capacity, serving as a surrogate marker for clinical severity of PA. Conclusion: Our data illustrate that PA in IIM is associated with alterations of the NK cell repertoire, suggesting a relevant contribution of NK cells in certain IIMs, which might pave the way for NK cell-targeted therapeutic approaches.

## 1. Introduction

Extra-muscular manifestations of idiopathic inflammatory myopathies (IIM) remain a major driver of morbidity and mortality [1]. Among these, pulmonary affection (PA) is abundant, with the reported prevalence ranging between 20 and 78% [2,3]. PA in IIM is characterized as a restrictive pulmonary pattern with nonspecific interstitial pneumonia and fibrosis [4]. Although the PA associated with IIM typically occurs during or after the disease onset, 13 to 38% of PA precedes the diagnosis of IIM, particularly in dermatomyositis (DM) patients, constituting a diagnostic challenge for the treating physicians [4].

In patients with anti-synthetase syndrome (ASyS), PA with interstitial lung disease constitutes a paradigmatic symptom in nearly 3 out of 4 patients, resulting in it having a significant role as a diagnostic criterion in addition to the myositis specific antibodies (MSA) [3,5,6]. PA is also commonly described in patients with ‘polymyositis’ (PM) and DM and also occurs in amyopathic variants of DM [3,7]. However, the specific interplay of the different immune cell populations in IIM with PA has not yet been fully elucidated, so that effective, but also selective, treatment approaches are currently lacking.

To understand the pathophysiology of IIM-associated PA, previous have studies highlighted the role of cytokines, since serum levels of proinflammatory interleukin (IL) 6 and IL 8, as well as tumor necrosis factor-alpha and interferon gamma were significantly increased in IIM with PA [8]. Extended cytokine production might lead to an inflammatory state within the lung parenchyma, resulting in the relevant recruitment of innate and adaptive immune cells and associated tissue destruction. In terms of cytokine release, the role of MSA as a significant driver is potentially supported by the promising results of B cell depleting therapies [9]. However, the sometimes unfavorable risk–benefit ratio among long-term B-cell-depleting therapies underpins the need for new, more selective immunotherapies [10], and which requires the identification of new treatment targets involved in PA development and propagation [3].

Besides their critical role in early host defense against microbes, innate immune cells modulate the activity of dendritic cells and B- and T-cells [11,12,13,14]. In IIM-associated PA, macrophages, and especially natural killer (NK) cells, are non-selectively activated and release proteolytic enzymes such as histidyl-tRNA-synthetase in the lung [15,16,17]. Such enzymes are immunogenic, including an enhanced interaction with cytokines, as well as a chemoattractant potential to recruit and activate further immune cells [15,17]. NK cells orchestrate the extent of inflammatory the response using the cytolytic properties in IIM capable of inducing bystander self-tissue damage [14,18,19]. Importantly, we previously reported a substantially increased numbers of NK cells in the lungs of ASyS patients with PA [18]. Similarly, reduced levels of NK cells in peripheral blood were related to a higher disease activity in DM [20,21], possibly as a result of an increased migration of NK cells into target tissues, such as muscles or the lungs [19]. Altered phenotypes of NK cells, with changed levels of cell-surface receptors, were observed in ASyS, pointing towards shared mechanisms driving autoimmunity in IIM [18]. Since subsets of NK cells differ in their functional properties and homing characteristics, the aim of this study was to provide a detailed phenotypic and functional characterization of NK cells in IIM patients with PA.

## 2. Material and Methods

### 2.1. Study Cohort

Twenty-four patients with IIM (ASyS: *n* = 13, DM: *n* = 10; PM: *n* = 1), according to both EULAR/ACR classification criteria [6] and with respect to the recommendations of the 239th ENMC international workshop [22], were included in our study between January 2017 and September 2019. At inclusion, existing immunotherapies, especially the dosage of glucocorticoids, were as low as possible and had been prescribed at a stable dose for at least 3 months. IIM patients were divided into two subgroups (*n* = 12 with PA, *n* = 12 non-PA). Patients with PA had previously diagnosed interstitial lung disease or other clinical or radiological signs of an affected lung function in the absence of competing causes (e.g., smoking) [2,3]. The disease duration of IIM patients was defined as the time in months between symptom onset and blood sampling. The antibody status was determined using a EUROLINE Blot assay (antibodies: Mi-2 alpha, Mi-2 beta, TIF1g, MDA5, NXP2, SAE1, Ku, PM-Scl100, PM-Scl75, Jo-1, SRP, PL-7, PL-12, EJ, OJ, Ro-52, Euroimmun, Lübeck, Germany). Creatine kinase (CK) levels were measured during routine laboratory testing at the same time as the performance of study blood sampling. Computer tomography (CT) was performed to assess lung involvement, as a diagnostic procedure in the clinical routine. CT scans were evaluated by two specialized radiologists. The individual findings are given in Table 1. For pulmonary function tests, percentage predicted vital capacity (%VC) and diffusing capacity of carbon monoxide (%DLCO) were obtained. For the clinical assessment, manual muscle testing of 8 muscle groups (MMT-8) was used.

The local ethics committee (2016-053-f-S) approved blood sampling, and all subjects provided written informed consent before entering the study. This trial was conducted in accordance with the Declaration of Helsinki.

### 2.2. Sampling and Flow Cytometric Analysis of Peripheral Blood Mononuclear Cells

Whole blood samples were collected from study subjects. Afterwards, peripheral blood mononuclear cells (PBMC) were isolated by Ficoll (Sigma-Aldrich St. Louis, MO, USA) density gradient centrifugation and stored in liquid nitrogen, according to our standard operating procedure (SOP) until usage [23]. Freshly thawed or stimulated PBMCs were centrifuged at 300× *g* for 5 min. Thereafter, PBMCs were resuspended in phosphate buffered saline (PBS, Sigma-Aldrich, St. Louis, MO, USA) supplemented with 2% heat-inactivated fetal bovine serum (FBS, GE Healthcare, Chicago, IL, USA) and 2 mM ethylenediaminetetraacetic acid (EDTA, Sigma-Aldrich, St. Louis, MO, USA) and incubated with fluorochrome-conjugated antibodies at 4 °C for 30 min. The staining of chemokine receptors was performed at 4 °C for 30 min. When indicated, PBMCs were washed and additionally stained with a Zombie NIR or Aqua Fixable Viability Kit (Biolegend, San Diego, CA, USA), according to the manufacturer’s manual, to distinguish between dead and living cells. PBMCs were washed and resuspended in PBS/FBS/EDTA, then analyzed by flow cytometry using a CytoFlex Flow Cytometer (Beckman Coulter, Krefeld, NRW, Germany). For staining of intracellular proteins (perforin and granzyme, Gr) components from a BD Cytofix/Cytoperm™ Kit (BD Biosciences, Franklin Lakes, NJ, USA) were used according to the manufacturer’s manual. The antibodies used for immune cell phenotyping including surface molecules on NK cells are summarized in Supplementary Table S1. For experiments measuring MFI, all samples were stained with the same antibody mix at the same time points. Data were analyzed using Kaluza Flow Cytometry Analysis software version 2.1 (Beckman Coulter, Brea, CA, USA).

### 2.3. Gating Strategy

Immune cell subsets in the peripheral blood were identified using the following markers: Lymphocytes: forward scatter (FSC) vs. sideward scatter (SSC), B cells: CD19^+^ CD3^−^ Lymphocytes, T cells: CD3^+^ CD56^−^ Lymphocytes, CD4: CD4^+^ CD8^−^ T cells, CD8: CD8^+^ CD4^−^ T cells, NK cells: CD56^+^ CD3^−^ Lymphocytes, CD56^dim^: CD56^dim^ CD16^+^ NK cells, CD56^bright^: CD56^bright^ CD16^−^ NK cells (Supplementary Figure S1A).

### 2.4. Statistical Analysis

Statistical Analysis was performed using GraphPad Prism 9.1 (GraphPad Software, Inc., San Diego, CA, USA; 10.01.2020). Data are presented as medians (IQR = interquartile range), means (standard deviation = SD), or *n* (%). Differences between groups were analyzed using an unpaired Student’s t test or Mann–Whitney-U test, as appropriate, or Kruskal–Wallis test for multiple groups. To account for multiple comparisons, statistical significance was corrected using the false discovery rate (FDR) approach, using a threshold of Q = 5%. For prediction of %VC and %DLCO, we employed a model of simple linear regression with the former as dependent variable. Goodness-of-fit is given as R squared (R^2^). Differences were considered statistically significant with the following *p*-values: * *p* < 0.05, ** *p* < 0.01, *** *p* < 0.001, and **** *p* < 0.0001.

### 2.5. Data Availability

All data associated with this study are present in the paper. The data assessed in this study are available from the corresponding author on reasonable request.

## 3. Results

Baseline demographics of the entire cohort and clinical data are given in Table 1. Mean (SD) age of IIM patients with PA (52.5 (12.5) years) did not differ from non-PA patients (56.8 (11.3) years; *p* = 0.384). PA was more common in female patients, without reaching statistical significance (male: 5/12 in PA group vs 10/12 in non-PA group; *p* = 0.089). Mean (SD) disease duration was 39 (62) months and did not differ between PA and non-PA patients (33 (34) months vs. 27 (44) months, *p* = 0.768). Median (range) MMT-8 score of the entire cohort was 142 (90–150) and revealed no difference between both groups (PA: 147.5 (122–150) vs. non-PA 141 (90–150), *p* = 0.492). %VC was 63.5 (23) for the total PA cohort and did not differ between the subgroups of ASyS and DM patients (ASyS 65 (32), DM 62 (20), *p* = 0.812). %DLCO of the total PA cohort was 47.2% (23) and did not reveal significant differences between ASyS and DM patients (ASyS 51 (47) DM 41 (27), DM *p* = 0.592). Of note, the %VC and %DLCO of non-PA patients were unremarkable. Mean (SD) creatine kinase (CK) levels were elevated in our cohort (2036 (3678) U/L), but unremarkable between both subgroups (PA 2127 (4065) U/L vs non-PA 1947 (3426) U/L, *p* = 0.907). We observed a wide range of MSA in our cohort, without a remarkable predominance within a distinct group (Table 1).

**Figure 1:**
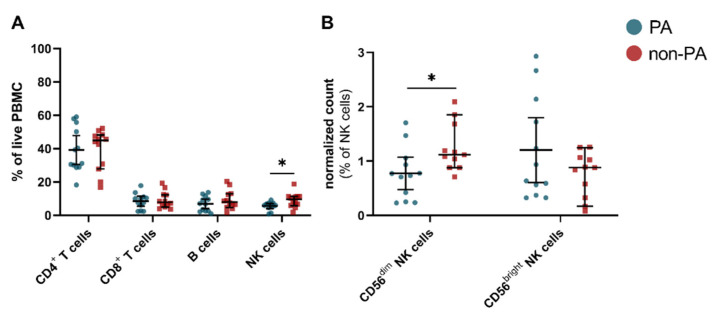
NK cell patterns in idiopathic inflammatory myopathies. (**A**) Relative cell numbers of leukocytes. (**B**) Relative cell numbers of CD16^+^ NK cell subsets after normalization. CD56^dim^ CD16^dim^ NK cells were excluded. Differences were determined by Mann–Whitney test for PA (blue) and non-PA (red). To account for multiple comparisons, statistical significance was corrected by the false discovery rate (FDR). A threshold of Q = 5% was used for FDR. Error bars display mean (IQR). Abbreviations: NK = natural killer, PA = pulmonary affection, PBMC = peripheral blood mononuclear cell * *p* < 0.05.

As the next step, we evaluated the distribution of immune cells in the peripheral blood using a flow cytometric approach. We did not observe any differences for B cells, CD4^+^, and CD8^+^ T cells, while the total NK cells in IIM patients with PA were reduced compared to the non-PA IIM group (Figure 1A). Notably, changes within the CD16^+^ NK cell compartment in IIM patients with PA were driven by a particular decrease of CD56^dim^ NK cells, while CD56^bright^ NK cells remained unchanged (Figure 1B).

**Figure 2:**
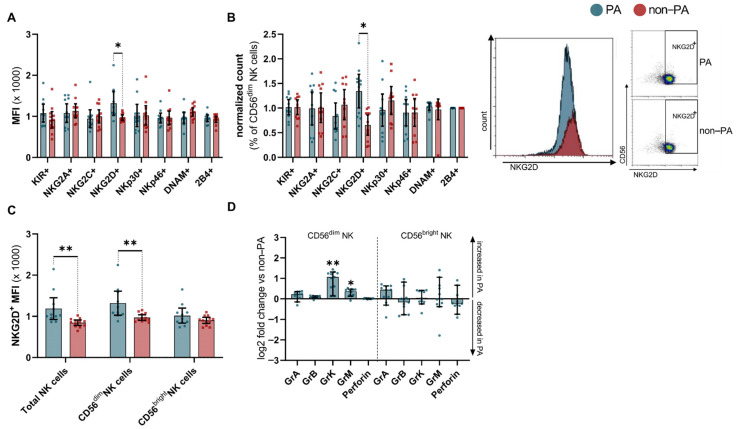
NK cell receptors are altered in IIM patients with pulmonary affection. (**A**) Normalized mean fluorescence intensity of NK cell surface receptors. (**B**) Relative cell number of CD56^dim^ staining positive for the respective NK cell surface receptor (left), and representative plots displaying differences of PA and non-PA (right). (**C**) MFI of NKG2D^+^ on total NK cells and NK cell subsets, respectively. Differences were determined by Mann–Whitney test for PA (blue) and non-PA (red). (**D**) Relative cell numbers of CD56^dim^ and CD56^bright^ NK cells from PA patients expressing corresponding granzyme or perforin compared to non-PA. Statistical analysis was performed using Kruskal–Wallis one-way analysis of variance with Dunn multiple comparison test. Error bars display mean (IQR). To account for multiple comparisons, statistical significance was corrected by the false discovery rate (FDR). A threshold of Q = 5% was used for FDR. Abbreviations: Gr = granzyme, MFI = mean fluorescence intensity, NK = natural killer, PA = pulmonary affection ** *p* < 0.01, * *p* < 0.05.

To further characterize NK cells from IIM patients with or without PA, we performed an extensive analysis of cell surface molecules. Expression of targets of interest was quantified using the median fluorescence intensity (MFI) and compared to non-PA samples. NK cells from IIM patients with PA were indistinguishable from those without lung involvement, in terms of the cell surface expression of a large panel of NK cell receptors (Supplementary Figure S1B). Of note, the proinflammatory receptor molecule NKG2D^+^ was abundantly expressed on NK cells from patients with PA compared with non-PA patients (Figure 2A). In line with this, the relative cell number of CD56^dim^ NK cells positive for NKG2D^+^ was increased in PA patients (Figure 2B). In-depth analysis of NKG2D^+^ NK cells revealed a pronounced increase in the CD56^dim^ subset, while levels of NKG2D^+^ on CD56^bright^ NK cells did not differ from the non-PA samples (Figure 2C).

Aiming to further dissect the cytolytic potential of the NK cell repertoire, we performed intracellular staining for serine proteases. We analyzed the number of cells with positive staining for intracellular granzymes (Gr) and perforin, as compared to non-PA samples in CD56^bright^ and CD56^dim^ NK cells. Here, CD56^bright^ NK cells displayed no relevant alterations, while the CD56^dim^ subset GrK and GrM were increased (Figure 2D).

**Figure 3:**
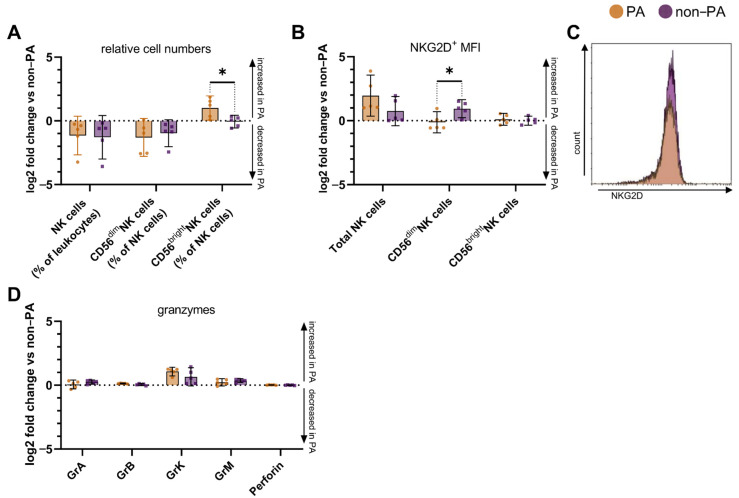
NK cell pattern of ASyS and DM patients with pulmonary manifestation. (**A**) Relative cell numbers from PA patients compared to non-PA. (**B**) MFI of NKG2D^+^ on total NK cells and NK cell subsets from PA patients compared to non-PA. (**C**) Representative plot displaying histograms for NKG2D on CD56^dim^ NK cells. (**D**) Relative cell numbers from PA patients expressing corresponding granzyme or perforin compared to non-PA. Differences were determined by Mann–Whitney test for DM (orange) and ASyS (purple). Error bars display mean (IQR). To account for multiple comparisons, statistical significance was corrected using the false discovery rate (FDR). A threshold of Q = 5% was used for FDR. Abbreviations: ASyS = anti-synthetase syndrome, DM = dermatomyositis, Gr = granzyme, MFI = mean fluorescence intensity, NK = natural killer, PA = pulmonary affection * *p* < 0.05.

We compared NK cell pattern of ASyS and DM patients presenting with PA. Interestingly, the fold change in the number of CD56^bright^ NK cells of PA compared to non-PA was more pronounced in DM compared to ASyS (Figure 3A). In contrast, the change in NKG2D^+^ levels on CD56^dim^ NK cells was increased in ASyS compared to DM, while other cell types displayed no relevant differences (Figure 3B,C). Analysis of intracellular serine proteases revealed no differences between ASyS and DM patients suffering from PA (Figure 3D).

**Figure 4:**
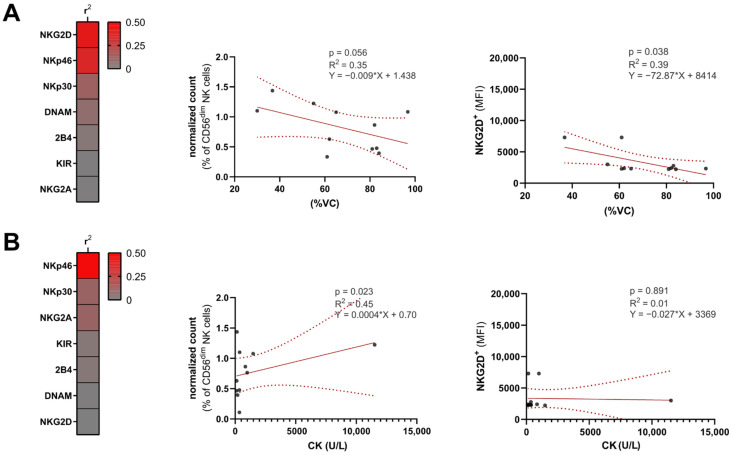
NK cell receptors are associated with severity of lung involvement. (**A**) Heatmap displaying R^2^ values of a linear regression model with %VC serving as the dependent variable and NK cell receptors as the influencing variable. For regression analysis, relative cell numbers were used for NKp30 and Nkp46, as these receptors are constitutively expressed upon inflammatory stimulation. Linear regression displaying the correlation of NKp46^+^ and NKG2D^+^ MFI with %VC, respectively. (**B**) Heatmap displaying R^2^ values of a linear regression model with serum CK serving as the dependent variable and NK cell receptors as the influencing variable. Linear regression displaying correlation of NKp46^+^ and NKG2D^+^ MFI with serum CK, respectively. Abbreviations: CK = creatine kinase, MFI = mean fluorescence intensity, NK = natural killer, %VC = percentage predicted vital capacity.

Lastly, we aimed to analyze the influence of NK cell surface receptor levels on clinical severity. Analysis of goodness-of-fit by measuring R^2^ revealed the NKG2D surface levels on CD56^dim^ NK cells to be robust predictors of VC, while other NK receptors displayed no meaningful correlation with VC (Figure 4A). For correlation analysis of NKp30 and NKp46, we used relative cell numbers, as these receptors are constitutively expressed upon inflammatory stimulation. The percentage of NKp46-positive cells correlated inversely with VC. In contrast, DLCO was not predicted by either NKp46 or NKG2D (NKp46 R^2^ = 0.15, *p* = 0.18; NKG2D R^2^ = 0.09, *p* = 0.57). Concurrently, we analyzed the association between serum CK and NK receptor levels (Figure 4B). Here, R^2^ values were obtained for NKG2D and NKp46; however, without reaching significance in the simple linear regression model.

## 4. Discussion

Lung involvement amplifies the burden of disease imposed by IIM [24]. However, a lack of pathophysiologic understanding precludes the development of effective diagnostic and therapeutic approaches to PA. Long considered as relatively simple cells, the status of NK cells has evolved into a diverse and versatile cell subset capable of pro-inflammatory and immunoregulatory effects [25]. Given that we previously observed a substantially increased number of NK cells in the lungs of ASyS patients with PA [18], we aimed to dissect the peripheral NK cell repertoire and improve our understanding of the elusive role of these cells in IIMs.

With regard to the clinical severity of muscle involvement, we found no difference between patients with or without PA. This is in line with previous data, demonstrating that involvement of the pulmonary compartment is not necessarily associated with the general severity disease course in IIM patients [24,26,27]. However, extra-muscular involvement results in the need for intensified immunotherapy, so that clinical or laboratory differences might have been masked, potentially limiting the prognostic use of such markers, in terms of extra-muscular involvement.

In the peripheral blood, we observed a decrease of peripheral NK cells, driven by a decrease of the CD56^dim^ NK cell subset [28]. Current understanding of NK cell phenotypes suggests that both CD56^dim^ and CD56^bright^ NK cells represent mature subsets, with distinct functional properties. CD56^dim^ NK cells represent a more terminally differentiated phenotype that may arise from CD56^bright^ NK cells. As such, CD56^dim^ NK cells express higher levels of CD16 and granzymes and are potent mediators of cytolysis and antibody-dependent cellular cytotoxicity [29,30].

In line with this observation, a number of studies have previously detected reduced levels of peripheral NK cells in autoimmune disorders, such as lupus erythematosus [31] or multiple sclerosis [32,33]; a behavior potentially attributed to NK cell infiltration into inflamed tissue [34]. However, it should be noted that this viewpoint is contested, and even within the same disease, NK cell function and distribution appear to differ substantially; either promoting or protecting against tissue damage [25,35]. Our data, observing a particular decrease of peripheral cytotoxic NK cells, supports the view of NK cell infiltration to inflamed tissue. This notion is further supported by previous observations of the considerably increased NK cell numbers in the lungs of ASyS patients with PA compared to non-diseased controls [18,19,36].

The investigation of surface NK cell receptors demonstrated an upregulation of NKG2D for PA patients, notably on CD56^dim^ cells. Abundantly expressed on NK cells, NKG2D acts as a sensor for stressed cells [37]. Ligation of NKG2D by self-proteins results in the release of cytokines and the stimulation of cell-mediated cytotoxicity [38,39,40]. Interestingly, chronic exposure to NKG2D ligands results in downregulation of this receptor and, consequently, reduced NKG2D-mediated cytotoxicity [35,41]. Of note, NKG2D modulates the activation threshold for NK cells via the NKG2D–DAP12 signaling axis, leading to upregulation of the adaptor CD3ζ chain and the tyrosine kinase, ZAP-70 [42]. Stimulation of airway epithelial cells results in upregulation of NKG2D ligands (major histocompatibility complex class I chain-related B and the UL-16 binding protein ligands) pointing towards a potential mechanism by which lung inflammation promotes pulmonary immune cell activation [43]. Interestingly, other ‘classical’ activation receptors such as NKp30 or NKp46 remained unchanged in PA, leading us to hypothesize that altered NKG2D signaling might regulate NK cell homeostasis in PA, particularly as NKG2D expression correlated with the level of lung involvement. This view of enhanced NK cell activation in IIM with PA is supported by the accumulation of CD56^dim^ NK cells staining positive for serine proteases capable of mediating cytotoxicity, such as GrB or GrM. In this context, GrM is of particular interest, as this protease has been shown to modulate cytokine release, neutrophil infiltration, and more severe histopathologies in models of inflammation, such as ulcerative colitis or endotoxemia [42,44].

While the NK cell patterns were mostly indistinguishable between disease subtypes, CD56^bright^ NK cells were increased in DM compared to ASyS. With regard to the NKG2D levels, the increase of this surface receptor was mostly driven by ASyS patients, pointing to diverging NK cell patterns in IIM.

The importance of NK cells in relation to the severity of lung function is supported by the fact that we found an association between NKG2D^+^ and NKp46^+^ cell surface levels and reduced %VC in patients with PA, while the CK levels in peripheral blood did not show correlations with NK cell patterns. Consequently, this underpins the role of NK cells in the pathophysiology of lung involvement in IIM patients and points to potential surrogate markers for predicting pulmonary damage.

Regarding potential limitations, in particular due to the small sample size, we acknowledge that this study did not aim to deduce a causality between the observed NK cell alterations and lung involvement. Further studies, possibly employing emerging murine IIM models [45], are needed to provide further mechanistic insights and guide the development of treatment strategies. Furthermore, the scope of this study did not include attempting to characterize the subset of CD56^dim^ CD16^dim^ NK cells, providing a potential caveat. Moreover, although patients were paired and we accounted for clinical and demographic characteristics, IIM present as a heterogeneous group, and individual differences might lead to bias. For instance, anti-MDA-5-positive DM patients are characterized by a rapidly progressive PA, in contrast to other DM subtypes. Notably, our cohort comprised only one patient with anti-MDA-5 antibody. Immunosuppressive therapies were more frequent in the PA cohort, given the clinical severity. However, the NK cell repertoire is mostly resistant to immunosuppressive drugs, including cyclophosphamide [46], making therapeutic bias less likely.

Nevertheless, we believe that this study shines a spotlight on the elusive and often underappreciated role of NK cells in autoimmunity. Understanding the mechanism by which NK cells initiate, propagate, or modulate tissue specific consequences of IIM might enable us to form a more conclusive concept of autoimmunity in IIM. Altered NKG2D signaling as a master regulator [38] of immune cell activation is of particular interest and warrants further mechanical studies.

## Figures and Tables

**Table 1:** Demographics and baseline disease characteristics. Abbreviations: AZA = azathioprine, CK = creatine kinase, CYP = cyclophosphamide, CyS = cyclosporine, F = female, IVIG = intravenous immunoglobulins, M = male, MMT-8 = manual muscle testing of 8 muscle groups, MTX = methotrexate.

Patient	Age	Sex	Diagnosis	Antibody	Known Malignancy	CK Level (U/L)	Characteristics of PA	Treatments
1	54	M	ASyS	JO1	-	1046	none	MMF, steroids
2	43	M	ASyS	JO1	-	249		IVIG, steroids
3	49	M	DM	MI-2	-	2422		none
4	53	M	PM	negative	-	1588		IVIG
5	43	M	DM	NXP2	oropharyngeal	4576		steroids
6	66	F	DM	negative	-	11,980		none
7	68	M	DM	negative	colorectal	99		MTX, steroids, IVIG
8	51	F	DM	TIF-1	-	61		AZA
9	79	M	ASyS	PL7	-	243		none
10	48	M	ASyS	negative	-	580		steroids
11	68	M	DM	TIF-1	-	399		none
12	60	M	ASyS	PL12	-	115		none
13	41	M	ASyS	PL7	-	355	pulmonary fibrosis with diffusion restriction	CYP, steroids
14	37	M	ASyS	JO1	-	354	alveolitis, pulmonary fibrosis	CYP, steroids
15	56	F	ASyS	EJ	-	845	usual interstitial pneumonia	CYP, steroids
16	63	F	DM	TIF-1	-	204	emphysema, pulmonary fibrosis with diffusion restriction	CyS, steroids
17	60	F	ASyS	JO1	-	165	pulmonary fibrosis with diffusion restriction	IVIG
18	76	F	DM	MI2	-	1489	pulmonary fibrosis with diffusion restriction	AZA
19	46	M	DM	MDA5	-	110	pulmonary fibrosis with diffusion restriction	CyS
20	47	F	ASyS	JO1	-	10,000	pulmonary fibrosis with diffusion restriction	MTX
21	46	M	ASyS	PL7	-	11,520	pulmonary fibrosis with diffusion restriction	Steroids
22	35	F	DM	SSA	-	373	pulmonary fibrosis with diffusion restriction	steroids, IVIG
23	57	M	ASyS	PL7	-	150	pulmonary fibrosis with diffusion restriction	AZA, steroids
24	66	F	ASyS	PL7	-	373	interstitial pneumonia	MTX

## Data Availability

Not applicable.

## Supplementary Materials

The following are available online at https://www.mdpi.com/article/10.3390/cells10102551/s1, Figure S1. Gating strategy, Table S1. Antibodies used for flow cytometric analysis of PBMCs.
