# Supplementary material for: NK Cell Patterns in Idiopathic Inflammatory Myopathies with Pulmonary Affection
Source: Cells. 2021 Sep 27;10(10):2551. doi: 10.3390/cells10102551 (PMC8534165; doi:10.3390/cells10102551)
Supplement: Supplementary file 1 [file cells-10-02551-s001.zip › cells-1364838-supplementary.pdf]

Table S1. Antibodies used for flow cytometric analysis of PBMCs.

Fluorochrome	CD No. Name	Clone	Company	Order No.
FITC	**pan-KIR: CD158a/h, b/j, e1/2, i**	11PB6	Miltenyi	130-099-209
FITC	**Granzyme K**	GM6C3	SantaCruz	sc56125-FTIC
PE	**CD56**	N901	Beckman Coulter	A07788
PE	CD159c/NKG2C	REA205	Miltenyi	130-103-635
BV785	CD16	3G8	Biolegend	302046
ECD	**CD20**	B9E9	Beckman Coulter	IM3607U
PerCP/Cy5.5	**CD226 DNAM-1**	11A8	Biolegend	338314
PC7	**CD159a/NKG2A**	Z199	Beckman Coulter	B10246
PECy7	**Granzyme A**	GzA-3G8.5	eBiosciences	25-9177-42
APC	**CD3**	Hit3a	Biolegend	300312
BV510	**CD4**	OKT4	Biolegend	317444
BV605	**CD45RO**	UCHL1	Biolegend	304238
APC	**CD336/NKp44**	P44-8	Biolegend	325110
eFluor660	**Granzyme M**	4B2G4	eBiosciences	50-9774-42
APC	**CD336/NKp44**	P44-8	Biolegend	325110
APC-A700	**CD8**	B9.11	Beckman Coulter	PN A66332
PE	**Granzyme B**	REA226	Miltenyi	130-101-351
APC-A700	**NKG2D**		R&D	FAB139N-100
APC/Cy7	**CD244/2B4**	C1.7	Biolegend	329518
PacB	**CD57**	HCD57	Biolegend	322316
VioBlue	**Perforin**	REA1061	Miltenyi	130-096-671
Biotin	**CD337/NKp30**	AF29-4D12	Miltenyi	130-092-553
BV650	**CD335/NKp46**	9,00E+02	Biolegend	331927

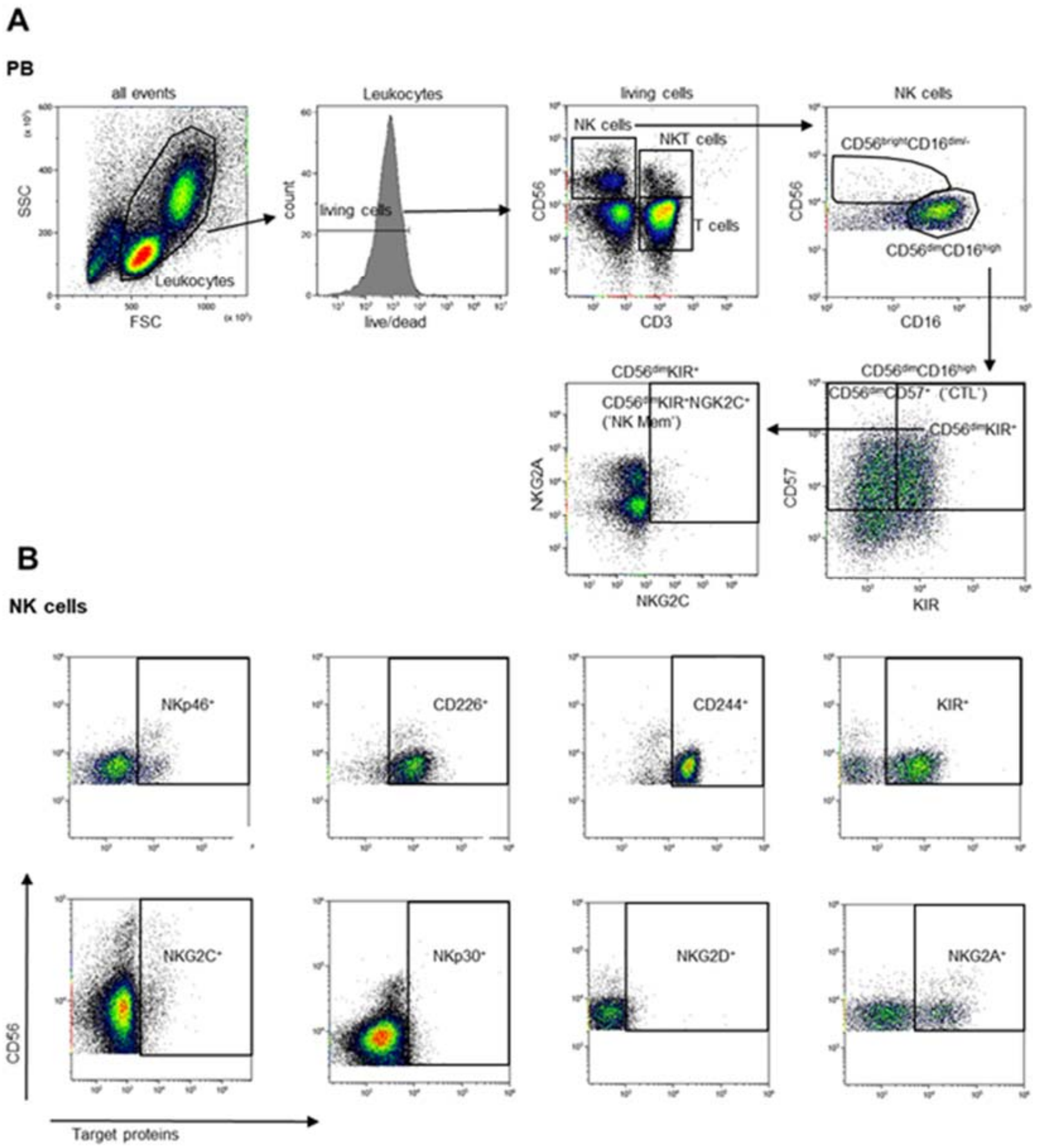

Figure S1. Gating strategy. (**A**) Single cells from the peripheral blood (PB) were simultaneously analyzed by flow cytometry. Total leukocytes were identified by forward scatter (FSC) and sideward scatter (SSC). Live/dead discrimination was determined using the amine reactive dye Aqua. From these cells, we identified lymphocyte subsets based on CD3 signal and CD56 expression. Next, we selected natural killer (NK) cell subsets based on CD56 and CD16 expression. $CD56^{dim}CD16^{high}$ were further differentiated based CD57 und KIR expression into cytotoxic T cell subsets (CTL) and memory NK cells (memory NK). (**B**) NK cells, $CD56^{bright}CD16^{dim/-}$, $CD56^{dim}CD16^{high}$ CTL and memory NK cells were differentiated based on their surface marker expression in NKp46, CD226, CD244, KIR, NKG2C, Nkp30, NKG2D and NKG2A positive cells (here exemplarily shown at total NK cells).
